# Extra Supplement—The Nursing Mirror

**Published:** 1890-07-26

**Authors:** 


Being the Extra Nursing Supplement of "The Hospital" Newspaper.

All Contributions for this Supplement should be addressed to the Editor, The Hospital, 140, Strand, London, W.C., and should have the word "Nursing" plainly written in left-hand top corner of the envelope.

### En Passant.

**Letters of Thanks.**—We are much pleased and gratified by the large numbers of letters of thanks (some in verse!) sent us by members of the "First Thousand," but circumstances preclude the possibility of our publishing these letters. We hope we expressed the thanks of all to all very fully in our number of July 12th, which was specially enlarged for the purpose. Our ordinary pages cannot contain all the letters and verses we have received on the subject of the reception at Marlborough House.

**Nurses Wanted.**—Two of our advertisements this week are for ten nurses, so that there can be no lack of work for women with a hospital training. Another notable point which struck us last week in glancing over the advertisements was a demand for male nurses. We women are sometimes inclined to be as unfair to male nurses as doctors are to female doctors. Male nurses have to pick up their training as they can, and certainly deserve a better recognition than is vouchsafed to them. Another point for rejoicing is the demand for a trained assistant matron for Maidstone Asylum, and trained nurses for Brookwood Asylum. Shall we in time achieve our heart's desire of securing the right of the dying lunatic to be properly nursed? The report of the Philadelphia Hospital states: "During the year our nurses have been introduced into every ward of the women's department of the insane hospital, and with very satisfactory results." We ought not to fall behind America.

**Noble Lords on Nursing Details.**—It is most amusing in the House of Lords' committee-room to hear how some of the Committee return again and again to trivial points. The lamps and inkstands, which the nurses of the London Hospital have to polish, are one of the things the noble lords cannot understand. In vain they are told that the lamp is not primarily for lighting the ward, but for carrying round with the surgeon and for use in ophthalmic or throat cases, and therefore more or less of a surgical instrument. As such, it is of course the care of the nurse; to keep a lamp clean and sweet is always a difficulty, even in private houses. Servants never will give proper time and attention to lamps, and put as much oil out as they put inside them as a rule. A good housewife of the middle-classes always does her own lamps, and a good nurse would certainly never permit a ward-maid to interfere with her surgeon's lamp. Both lamps and inkstands being pewter, and the inkstand also being for the use of the surgeon, it is only natural that the nurse should clean the inkstand when cleaning the lamp. Not only this, my lords, but the nurses delight in polishing and keeping bright the strapping tins, hot-water tins, &c. With regard to the hours the nurses work and their short holidays, there is room for much improvement. We give this week, some questions being asked now in Australia, where a somewhat similar inquiry into charities is being made. There the hours of the nurses are even longer than here. Mr. Treves held forth to the Committee on the subject of flat-foot and proper nursing shoes; we wonder if Mr. Treves has ever seen Parker's "ward shoes," which are strong enough to support the instep, and yet pliant enough to be comfortable? They are also noiseless. It is pleasant to see how thoroughly Lord Sandhurst, the Chairman of the Committee, is up in all hospital details; his interest in each question is practical, and his patience seems unbounded.

**A Home for Epileptics.**—One of our correspondents earnestly appeals for the name of some home where a girl suffering from epilepsy could be received for about 6s. a-week. This query has met with no response from our readers, so we bring it forward here in larger type, hoping it may catch the eye of someone who knows where such a home can be found. Our readers would greatly oblige by sometimes answering, as well as asking, queries.

**Mary Adelaide Nurses.**—Mrs. Bonham-Carter writes the seventeenth quarterly letter to the Mary Adelaide Nurses, and gives a most interesting account of the rise and progress of trained nursing. The tale of Florence Nightingale is an oft-told tale, but ever causes a woman's heart to burn with admiration and fervent longing to follow such a noble example of sweetness and strength. Some less-known facts come forth in Mrs. Bonham-Carter's pamphlet, which is certainly the best of these letters we have ever seen. The labours of Miss Agnes Jones, Miss Torrance, Miss Annie Hill, and other pioneers of nursing are referred to. Arthur Hugh Clough, the poet, was the first secretary of the Nightingale Fund, and some beautiful verses of his—"Say not the struggle nought availeth, The labour and the wounds are vain," conclude the letter.

**Short Items.**—Last Saturday's *Queen* contained further sketches of nursing costumes.—Dr. Bond, of Leicester, is engaged to Miss Simpson, late nurse; Dr. Openshaw, of the London, is engaged to Miss Pratt, late sister.—Nurse Emily Walker, Nurse Tilyard, and Nurse Caroline Walker, have taken the three prizes given annually to the probationers of the London Hospital.—Mrs. Scharlieb's daughter has taken a scholarship at University College; is it to be a case of Miss Fawcett again?—American Deaconesses have to train two years, and must have at least three months of hospital service.—Nurse Foley collected over £11 at Richmond on Hospital Saturday.—Miss Philippa Hicks is going abroad for her health.—The gallery at the Garrick Theatre is as good as the upper boxes at the Globe, and "A Pair of Spectacles" is an amusing comedy if nurses care to spend their shillings on an evening's laughter.

**Trained Male Nurses.**—In the end of June, General Sir C. E. Gordon, C.B., presided at the annual meeting of the Hamilton Association for Providing Trained Male Nurses, which was held at 57, Park Street, Grosvenor Square. The report stated that the financial outlook was much more favourable than at the corresponding period of last year. During the twelve months the loss had only amounted to £61, as compared with £300 and £400 in previous years, and this loss had been more than defrayed by the subscriptions and donations received during the year. The training of men as nurses was a branch of the work in which an advance had been made during the year, and an experiment had been made of accepting new candidates as "probationers" only in the first instance, and making them serve for a time on trial before finally enrolling them. Fifty men were on the books of the association on May 31st, 1890, and of this number thirty-five were remaining in employment at that date. The receipts for the year amounted to £3,482, and after payment of expenses, £200 had been placed on deposit at the bank, carrying forward £267. In moving the adoption of the report, which was seconded by Dr. Prosser James, and carried, the Chairman remarked that the association had made steady progress during the past year, and that it might now be considered as permanently established. Sir W. Mackenzie also addressed the gathering.

### Sketches of Insanity.

By Francis H. Walmsley, M.D. (Senior Assistant Medical Officer of Leavesden Asylum).

### IV.—THE ACTS OF THE INSANE.

The acts of the insane are generally connected with their delusions, though frequently it is difficult to trace the association. It is probably beyond the power of the sane mind to conceive the confusion which reigns in the mind of the madman. There is no action, however absurd, no crime, however atrocious, that his delusion cannot create, prompt, and justify. Thus a religious maniac kills a relative or an attendant, imagining him to be a fiend. Others believe themselves commanded by spirits to commit outrageous acts. Fathers and mothers kill their children under the pressure of domestic anxiety, culminating in an insane dread of starvation. Others, again, think they have a direct commission from the Deity to fulfil some mission of wrath or extirpation.

The delusions of the insane generally pertain to self, and their morbid self-feeling is revealed in their states of depression and exaltation, in their fear and their fury.

Broadly speaking, we may say that in the mentally deranged there is manifested a condition of over-action or under-action of the whole organism and of its parts; in the former condition—characterised by exaltation and exhilaration—there is an excessive generation of nerve energy, accompanied by a rapid and continuous discharge, often in a sudden and violent form, giving rise to sustained excitement with excessive mental and motor activity. In the condition of under-action the generation and diffusion of nerve force is scanty, hence we have depression and gloom with appropriate acts and conduct—despondency, torpor, and inactivity.

The analogy already pointed out between dreaming and insanity extends to some cases, at least, of somnambulism. The following description will aid us in our efforts to interpret the uncontrollable impulse prompting and directing the acts of the homicidal lunatic: Late one evening a monk entered the room of the prior of the convent, his eyes open and fixed, a frown on his features, and a knife in his hand. He walked straight up to the bed, as if to ascertain if the prior were there, and then gave three stabs which penetrated the bedclothes and a mat that served the purpose of a mattrass. He then returned with his features relaxed, and wearing an air of satisfaction. The next day, on being questioned, he confessed that, having dreamed that his mother had been murdered by the prior, and that her spirit had appeared to him and cried for vengeance, he was transported to fury, and ran directly to stab her assassin. Shortly after, he awoke, covered with perspiration, rejoiced to find that it was only a dream.

The acts of the lunatic often evince the same forethought and preparation as those of the sane, as is seen in the case which recently occurred in Chicago, where a priest was shot, and it was feared fatally wounded, by a young man supposed to be in a dying condition, to whom the priest was about to administer the last sacraments. It was subsequently discovered that the man was insane, and in no immediate danger of dying.

Patients suffering from melancholia frequently betray an irresistible propensity to self-destruction. Suicide is often the result of a sudden impulse, on very slight provocation. Some patients, interpreting too literally the Scriptural injunction, will mutilate their bodies: will destroy the eye or sever the hand from the arm. Certain patients have the delusion of double personality, and will hold conversations with a second self within them, will quarrel, and end by striking severely the *left* face, nose, and eye with the *right* hand.

An individual having become low-spirited and despondent by reason of his father's death, developed the delusion that it was possible to communicate with his spirit. He posted a letter containing a message to his father, and attempted to set fire to the box, believing that if the letter was consumed the smoke would be wafted away and convey the message to heaven.

An epileptic, seized with a sudden paroxysm, fell in a shop, got up, and, eluding the shopman and his friends, ran away, leaving his hat and order-book behind. He was discovered a quarter of a-mile away, asking for his hat at all the shops, but not having recovered his senses; nor did he become conscious until he got to the railway, ten minutes after. He was in the condition known as the "epileptic dream," or the state of unconscious cerebral automatism.

*(To be continued.)*

### The Training of Nurses in France.

### HOSPITAL ADMINISTRATION.

The second volume of the *Manuel Pratique* deals with hospital administration, and is written by Edouard Pinon, superintendent of *La Pitié*. The opening chapter tells of the formation of the present system of nursing by the municipal council of Paris, who, in 1877, sanctioned the proposals of Dr. Bourneville for training nurses, both male and female, in the Government hospitals. The work was begun at Salpetriere and at Bicêtre that same year, and the sisters of charity were gradually replaced throughout the chief hospitals by lay nurses. The course of training lasts a year, and the nurses have to attend theoretical and practical classes. The theoretical course includes seven subjects: Hospital administration, anatomy, physiology, minor surgery and dressings, hygiene, pharmacy, and monthly nursing. These classes are held twice a week from eight to nine in the evening. Each lecturer gives eighteen lessons, and demands of his hearers essays on two given subjects. The practical classes are held almost daily. A diploma is awarded to the successful pupils at the end of the year. The laicitation, or replacing of nuns by nurses, took place at the following dates at the Parisian hospitals: Salpetriere and Bicêtre 1877, Laennec 1878, La Pitié 1880, La Rochefoucauld, Des Menages, and St. Antoine, 1881; Lourcine and Tenon 1882, the Incurable Hospital and Cochin 1885, the Necker, Enfants-Assistés, and Enfants-malades in 1886; the Trousseau, Lariboisière, and Beaujon in 1887; and La Charité in 1888. The later dates are not given, but the work went steadily on till last year, when the sisters of charity were expelled from their last posts. Surely it is proof that the lay nurses are not so bad as the Catholic papers paint them, that they have so gradually, yet so surely, superseded the sisters. The surgeons of Paris would never have continued to encourage the introduction of lay-nurses if they found them less efficient than the *sœurs de charité*. A good many of the pages of the volume before us are filled with tales of the origin of hospitals and their formation in the old Roman days. One chapter deals with the daily life within the hospital wards and the various printed reports, which are similar to those used in most London hospitals. The diet tables are very liberal; the morning always begins with *soupe maigre*; there is a hot meat meal in the middle of the day, and hot fish or soup with vegetables for supper. Wine is allowed where we allow beer. The afternoon seems to be the time when the heavy extra duties are done, the male nurses are then bidden to polish the floors, the female nurses to scrub the lockers and tables. The seventh chapter deals with the moral qualities of the nurses, and is of special interest. First of all is a discourse on cleanliness: "Cleanliness is one of the most necessary virtues of a nurse. She herself must be clean as well as everything around her—the patients, sheets, curtains, beds, utensils, windows, floors, and walls. In every ward a general turn-out ought to be made once a week; that is to say, that the walls and pillars are brushed, the window panes cleaned, the curtains shaken, the night stools, curtains, and quilts washed in potash water, and again rinsed in phenol water. It is the duty of the superintendent to see to all this in the afternoon, and to fix the most convenient day and time." The nurses are bidden not to take service lightly, or merely as temporary employment; the writer well insists that nursing is a serious calling, and only to be taken up as a permanency. The faults of the sisters of charity are defined as lack of docility to the doctors, absence of professional knowledge or training, and the desire to proselytise. During times of epidemic, it seems, the lay nurses have stuck to their posts with the same devotion displayed by the sisters. Finally, the nurses are bidden to be active and exact, good-humoured, obedient, and modest, not gossips and not coquettes, but attentive to the wants of their patients and to the teaching of their professors. This volume ends with short biographical notices of those whose names have been given to the wards of the different hospitals. There are several English and several feminine names, but notably absent is that of Florence Nightingale, presumably because she is still alive. Here are a few of the names: Bacon, Locke, Dr. Carette, Descartes, Helmholtz, Harvey, Jenner, Lavoisier, Lisfranc, Volney, and Voltaire!

### Notes from Australia.

(*By Our Own Correspondent.*)

THE chief events interesting to HOSPITAL readers during the month of May have been the proceedings of the Charity Commission; the continual friction at the Women's Hospital; the opening of the Home of Hope by the Earl and Countess of Hopetown; the Countess of Hopetown's visit to the Austen Hospital and to the Women's Convalescent Home, where she was courteously received in each case. At one of the Charity Commission meetings Mr. Langton said:—

"I believe in having women nurses solely. Their status should, if possible, be increased, and instruction should be given to pupil nurses." At another meeting the following conversation took place between the Chairman and Mr. Williams, Secretary for the Melbourne Hospital:—

The Chairman: What are your opinions with regard to the nursing arrangements at the hospital?

Mr. Williams said that it was necessary that the nursing should be done by women, and that they should be led by an educated person, who was a better nurse than any of them. The hospital should form a training school for nurses. There were 83 nurses altogether, and at the present time they received no special training in the shape of lectures. They were not engaged upon menial work except during the first year's service. The hours of work for a nurse were from seven a.m. till nine p.m., and they had half a day off each week.

The Chairman: Do you not think the hours rather long?

Mr. Williams: No; not for healthy women.

The Chairman: Do you think it is justifiable that these women should work 14 hours a-day?

Mr. Williams: We have not found it too great a strain.

The Chairman: That is not the point. Have they found it too great a strain?

Mr. Williams: We never had any complaints except once when some refractory nurses made the long hours a grievance, but though an opportunity was given them they declined to take advantage of it. The status of the nurses ought to be improved, and if there were funds available the committee would provide them with better accommodation at once. A proposal to introduce trained nurses into the institution was made about five years ago, but it met with great opposition. Since that time, however, the views of the management had changed with regard to the question.

These remarks should be fruitful, and probably will be, in making reforms for nurses. They are needed. It is a fact that in some instances men, not women, have been "nurses solely," *i.e.*, for all patients. It is quite a point gained that Miss Martelli, the new matron at Maryborough Hospital, has been allowed to advertise for a trained nurse for work in that hospital. One experienced and well-trained nurse is delighted with "Burdett's Hospital Annual"; she has been comparing statistics, and says where in England three nurses are required, we in Melbourne have one. Another nurse gives me the hours of work at the Alfred Hospital, which is certainly one, if not the best worked hospital in Victoria. The days are not all alike in the number of working hours; but for Class B the work hours amount to 80 for six days, one week, 78½ the alternate week. Sunday is a holiday. It says much for the truth of a Christian life that this nurse is "extremely happy in her work." But that is the more reason why governing bodies should be more careful of such useful women. We have but recently lost one of the best nurses in the Alfred Hospital, "such a good and faithful girl, beloved by all who knew her." She died from diphtheria; was overworked when overtaken by it, so had no power of resistance. Not so very long ago nine of the nurses had typhoid at the Sick Children's Hospital. Two of them died. During last month the new matron at the Sick Children's Convalescent Cottage had diphtheria. Of course there is always more or less danger in nursing, but more precautions should be taken for the nurses. Another item of present interest in Melbourne is how the appointment of medical officers shall be made.

### Everybody's Opinion.

[*Correspondence on all subjects is invited, but we cannot in any way be responsible for the opinions expressed by our correspondents. No communications can be entertained if the name and address of the correspondent is not given, or unless one side of the paper only be written on.*]

### THE NURSE.

B. J. E. WRIGHT writes: My attention having been drawn to the "lines" headed "The Nurse," in your useful paper, I shall feel obliged if you will insert a few words from me, who have recently been an in-patient in King's. I can hardly believe the writer has ever been in the hospital, for to apply such words as "pest," "scold," etc., to any of our nursing staff is a libel; he evidently lacks charity and gratitude. It is from remarks like these that complaints originate, which, when inquired into, are found to contain not an iota of truth. It must appear to everyone with ordinary sense that punctuality and regularity must govern all institutions.—[If any more of our readers want surgical operations performed to aid their sense of humour our medical officer is ready and willing. The lines in question were handed by the patient to the nurse of whom they were written, and sent by her to us. We solemnly assure B. J. E. W. that they are a joke.—*Editor.*]

### HOME OF REST.

NURSE M. B. writes: It is a pity there is no means taken by the originators of the proposed Home of Rest, to find out the opinion of the generality of nurses. Our friends mean so kindly, and the words to be said in favour of the project appear so many, that at first sight approval is given which a little more consideration would greatly qualify. A nurse must have a holiday, and it is needful that she should have rest both for body and mind. Pleasant cleanly rooms, well-ordered meals, and a little congenial society conduce to this end; also, expenses must be small, and where can these things be better combined than in a specially prepared Home? On the other hand, as remarked in these columns, nurses do not get a rest of mind by crowding together. Interesting as nursing subjects are to us, we should be able for a short time in the year to lie them on one side and open our minds to new impressions. It is true that we are only too prone to shut ourselves up in our profession, and to care little for the great social questions and needs that are discussed so eagerly outside. If our faculties were occasionally exercised by other affairs than those of the sick room, our sympathies would be wider, our interests increased, and we should be not only nurses, but companionable women. Hospital nurses can badly afford out of their poor pay the few weeks of sea or country air that is so necessary for them. They cannot always go to friends, and besides many of us feel that family details, more often than less, of struggle and hardship are not the most restful and invigorating things with which to preface another year of hard work. Yet, how to manage the doubled expenses that a time divided by loving duty and attention to health would entail? Still, I cannot think that a home is the best kind of help. Be it ever so well-managed there is a feeling of restraint on the part of the nurses; a sort of sensation that might be experienced by a privileged pauper. In a home of rest with which I am acquainted the tones of subdued deference in which the paying nurses speak to the sister-proprietress is most depressing, and utterly destructive both of comfort and fun. As far as bodily wants are concerned the place is perfect, but the visitors cannot apparently divest themselves of an overwhelming sense of obligation, which is quite needless, as their payments cover expenses; though I ought in justice to say that the profits cannot be great. I do not think the public should be appealed to on behalf of private nurses. The institutions and hospitals that take two-thirds of their earnings should certainly provide them with holiday expenses. The sooner the other London nursing homes follow the just example set by Great Ormond Street the better for their reputation and prosperity. Both the medical profession and the public are awakening to the fact that the private nurse has hitherto been made the source of large profit to others, and they will send to those places where she receives the largest proportion of her earnings.

"A Hospital Life Governor" must send name and address.

### The Nurse.

*(Another View by Another Patient.)*

Who is it comes at dead of night,
In dark blue gown, and apron white,
And gently asks if you're "all right"?
Our nurse!

Who is it then, with noiseless tread,
Goes softly round from bed to bed,
And shakes your pillow, lifts your head?
Our nurse!

Who is it keeps till morning light
A lonely, faithful watch all night,
Yet calls us with a smile so bright?
Our nurse!

Who, when the dim night hours are done,
And the day's busy work begun,
Is active now as anyone?
Our nurse!

Who's ever patient, cheerful, kind,
Will no unpleasant duty mind,
In whom a friend we always find?
Our nurse!

Who, when the day begins to wane,
Cheers us by her bright smile again
And robs the night of half its pain?
Our nurse!

A. G. C.

### Appointments.

Gibraltar Colonial Hospital.—Miss Aston has sailed for Gibraltar to undertake the matronship of this hospital. Miss Aston trained at St. Thomas's, was a sister there, and then assistant at Liverpool. For some time Miss Aston worked in Ceylon at Colombo Civil Hospital, and then returned to take the trying post of matron to Homerton Fever Hospital, which she had to resign this spring.

Hong Kong Government Hospital.—Miss Clara Eastmond has been appointed head sister to this hospital; she trained at Addenbrookes, and was night sister there for two years. Since 1885 Miss Eastmond has worked with great success as a sister of the London Hospital. Miss Emma Ireland, trained at St. Pancras and the London, and now sister there, and Miss Catherine MacIntosh, trained at the Liverpool Hospital for Women and at the London, are both going to Hong Kong with Miss Eastmond, to work under her as sisters of the chief wards. We heartily wish all three success in their venturesome undertaking.

Helensburgh Infirmary.—Miss Helen Spence entered on her duties as matron of the infirmary at Helensburgh on May 1st. She trained at the Seamen's Hospital, Greenwich, and the General Infirmary, Leeds, and was afterwards engaged in district nursing in Glasgow.

Halifax Borough Fever Hospital.—At a special meeting of the Sanitary Committee, held on Wednesday, Miss M. Robinson, of the Glasgow Fever Hospital, was appointed matron of the Borough Hospital out of 39 applicants. Miss Robinson has spent nearly ten years of her life in infectious hospitals, having been trained, and holding the certificate of the Nurses' Institution, Bradford. She afterwards took charge of the Moor Hospital at Newcastle during the small-pox epidemic, and for the last two years has been charge-nurse in the enteric wards of the Glasgow Fever Hospital, where she has had the care of all the diphtheria and tracheotomy cases. We congratulate Miss Robinson upon her promotion, which she has well earned, and wish her every success in her new appointments.

### Vacancies.

The following vacancies are announced:

### Assistant Superintendents.

Maidstone County Asylum; £85.
Nottingham Nursing Association; £60.

### Matrons.

Manchester Maternity Hospital; £50.

West of England Eye Infirmary, Exeter; £40.

### Head Nurse.

Southampton Fever Hospital; £40.

### Nurses.

Aberdeen Poorhouse; £22.

Brighton Children's Hospital; £25.

Bishop's Stortford Home; £25.

Blackheath Institution.

Beckett Hospital, Barnsley; £20.

Brighton Fever Hospital; £24.

Brixton Nursing Institution.

Bengeo Nurses' Home; £30.

Barony Parochial Hospital; £24.

Cheltenham Institution.

Chelsea Infirmary; £18.

Cardington Children's Hospital.

County of Cornwall Trained Nurses' Home; £25.

Children's Hospital, Manchester; £25.

Cumberland Infirmary (night); £24.

Chester Church Deaconesses' House; £24.

County Asylum, Devizes; £25.

Dinorwie Hospital (Nurse Matron).

East Greenwich Infirmary: £27.

General Nursing Institute.

Glasgow Fever Hospital.

Greenwich Union; £20.

Huddersfield Infirmary; £24.

Huddersfield Nurses' Home.

Isle of Wight Infirmary (night); £25.

Jersey Institute.

Kent Nursing Institution; £30.

Leicester Fever Hospital; £25.

Lowestoft Hospital; £20.

Mr. Wilson's Nursing Institution, Wimpole-street.

Middlesbrough Fever Hospital; £30.

Newcastle Nurses' Home.

North-Western Fever Hospital; £30.

Ophthalmic School, Hanwell; £26.

Poplar and Stepney Sick Asylum; £16 10s.

Sheffield Nurses' Home.

Sheffield Union; £20.

St. Saviour's Infirmary; £20.

Surgical Home, Devonshire-street; £25.

St. Helena Home.

Southampton Fever Hospital; £30.

St. Nicholas' Home, S.W.; £20.

Suffolk General Hospital; £20.

St. Leonard's, Shoreditch; £20.

Surrey County Asylum.

Victoria Home for Nurses, Bournemouth; £25.

Workhouse Infirmary Nursing Association.

### Male Attendants.

Darenth Asylum for Imbeciles; £25.

### District Nurses.

Barnsley Beckett Hospital; £20.

Boston Vicarage; £50.

Hulme District Nursing Institution; £25.

Jersey Institute; £25.

Manchester Sick Poor Institute; £25.

Nyrthfa, Brecon.

### Probationers.

Arbroath Infirmary.

Canterbury Nurses' Institute.

Central Ophthalmic Hospital.

Huddersfield Infirmary; £12.

Hull Royal Infirmary.

Hospital for Chronic and Convalescent Children, Highgate.

Kidderminster Infirmary.

Kidderminster Children's Hospital.

Kilmarnock Infirmary.

Lincoln County Hospital.

Leeds Nurses' Institute; £14.

Middlesbro' Fever Hospital.

Preston Royal Infirmary; Jubilee

Queen Victoria Nurses' Institute (Scotch branch).

St. Helena Home.

### Notes and Queries.

### Answers.

*Nurse A.*—To become an army nurse it is necessary to train for three years in a general hospital, and then apply to the Director General of the Army Medical Department, Victoria-street, Westminster. See the *Hospital Annual* for further particulars.

*Nurse E.*—Only those of the first thousand who joined the fund unassisted have a share of the special Bonus Fund of £5,000.

*F. W.*—You cannot get such a certificate, your testimonials must stand you instead. Read Lucke's "Lectures on Nursing," Kegan Paul, price 2s. 6d.

*Private Nurse.*—The certificates have been sent by post.

*Constant Reader.*—The pamphlet was given to a sufferer from sea-sickness after being reviewed, and the publisher's name is unfortunately forgotten. The person who became the possessor of the pamphlet was a stranger. We may mention, however, that all the principal points were duly noted in The Hospital. Dr. Dutton, the author, is at present absent from England travelling.

*Pension Fund Notice.*—Nurses can have their purses returned on application to the manager, 8, King Street, E.C., provided they were not amongst those selected by the Princess of Wales as a memento of the occasion.

### Amusements and Relaxation.

### SECOND QUARTERLY WORD COMPETITION.

Commenced July 5th, 1890, ends Sept. 27th, 1890.

The word for dissection for this, the FOURTH week of the quarter, being

"RASPBERRY."

| Names. | July 17th. | Totals. | Names. | July 17th. | Totals. |
|---|---|---|---|---|---|
| Dulcamara | 3 | 35 | Lightowlers | 2 | 30 |
| Tinie | 3 | 35 | Jenny Wren | 2 | 27 |
| Henri | 3 | 34 | Rhoda | — | 22 |
| Agamemnon | 2 | 33 | Annabel | — | 17 |
| Patience | 3 | 33 | Spare Moments | — | 19 |
| Brisbane | — | 28 | Qu'appelle | 3 | 33 |
| Nurse Hilda | 3 | 32 | | | |

